# Genetic determinants of arterial thrombosis in primary antiphospholipid syndrome: a systematic review

**DOI:** 10.3389/fimmu.2026.1761613

**Published:** 2026-03-05

**Authors:** Claudia Gavris, Silvia Sovaila, Laura Girdan, Adrian Purcarea

**Affiliations:** Facultatea de Medicină, Universitatea Transilvania din Brașov, Brasov, Romania

**Keywords:** arterial thrombosis, endothelial dysfunction, endothelial pathways, genetic polymorphisms and disease association, primary antiphospholipid syndrome (PAPS)

## Abstract

**Background:**

Primary Antiphospholipid Syndrome (PAPS) is a systemic autoimmune disorder characterized by arterial and/or venous thrombosis and obstetric morbidity. Arterial thrombosis, although less frequent than venous events, is associated with substantial morbidity and mortality. Alongside environmental and acquired factors, several genetic polymorphisms affecting coagulation, endothelial function, fibrinolysis, and platelet activation have been investigated in relation to thrombotic risk. Clarifying their contribution may help refine hypotheses for risk stratification.

**Objectives:**

To systematically review the available evidence on genetic polymorphisms associated with arterial thrombosis in PAPS and to evaluate their reported associations with arterial thrombotic manifestations.

**Search methods:**

Electronic searches were conducted in MEDLINE, the Cochrane Library, ClinicalTrials.gov, the GWAS Catalog, the Genetic Association Database, and Google Scholar for studies published up to November 2024.

**Selection criteria:**

Case–control, cohort, and genome-wide association studies were included if they assessed genetic variants in confirmed PAPS with arterial thrombosis. Comparators included healthy controls, patients with other autoimmune diseases, or APS without arterial events.

**Data collection and analysis:**

Two reviewers independently screened studies, extracted data, and assessed risk of bias using the Cochrane and ROBINS-I tools. Due to substantial heterogeneity in study design, populations, and outcome definitions, meta-analysis was not feasible, and results were summarized narratively.

**Main results:**

Eighteen studies published between 1994 and 2023 were identified, of which seventeen contributed to the genetic association synthesis. Variants in platelet membrane glycoproteins (GPIa 807T, GPIbα Kozak TC, and combined GPIa 807T plus GPIIb/IIIa PlA2) were the most frequently reported positive associations with arterial thrombosis. PAI-1 (4G) and MTHFR (C677T) showed inconsistent and weak associations, while the EPCR (PROCR) H1 haplotype was negatively associated with arterial thrombosis in isolated analyses. Classical thrombophilic mutations, including Factor V Leiden and Prothrombin G20210A, did not show consistent associations with arterial events. Overall certainty of evidence was low to very low.

**Conclusions:**

The currently reviewed evidence indicates that inherited susceptibility to arterial thrombosis in PAPS is more frequently linked to platelet-related, endothelial, and fibrinolytic pathways than to classical coagulation gene mutations. These associations are based on small, heterogeneous, and largely non-replicated studies and should be considered hypothesis-generating.

**Systematic Review Registration:**

https://www.crd.york.ac.uk/prospero/display_record.php?ID=CRD42024603974, identifier CRD42024603974.

## Highlights

- Evidence on inherited genetic susceptibility to arterial thrombosis in primary antiphospholipid syndrome is limited, heterogeneous, and largely derived from small observational studies.
- Classical genetic susceptibility to venous thrombosis does not show consistent associations with arterial thrombotic events in primary antiphospholipid syndrome.
- Reported genetic susceptibility is most frequently observed in platelet-related, endothelial, and fibrinolytic pathways, but lacks independent replication.
- Current findings should be regarded as hypothesis-generating and highlight the need for adequately powered, multicenter studies with standardized arterial outcome definitions.

## 1 Background

### 1.1 The antiphospholipid syndrome

Antiphospholipid syndrome is a systemic autoimmune disease characterized by inflammatory and thrombotic manifestations, including venous, arterial, and microvascular thrombosis, as well as obstetric morbidity. These clinical features arise from autoimmune and inflammatory processes driven by antiphospholipid antibodies, which play a central role in disease pathogenesis (1).

Primary antiphospholipid syndrome occurs in the absence of other autoimmune diseases, infections, drugs, or malignancies, whereas secondary antiphospholipid syndrome is most frequently associated with systemic lupus erythematosus (2). Additional autoimmune conditions and infectious or drug-related triggers have been reported in association with secondary antiphospholipid syndrome (3). Beyond the classical laboratory criteria, several non-criteria antiphospholipid antibodies have been described, although their clinical relevance remains incompletely defined (4, 5). The 2023 ACR/EULAR classification criteria reflect the multifaceted clinical and biological characteristics of antiphospholipid syndrome.

### 1.2 Clinical manifestations of antiphospholipid syndrome

Primary antiphospholipid syndrome manifests, with variable frequency, through thrombotic events involving both arterial and venous vascular territories, most commonly affecting cerebral arteries and the veins of the lower limbs (6). In addition to macrovascular thrombosis, antiphospholipid syndrome may involve microvascular disease affecting multiple organs, while catastrophic antiphospholipid syndrome represents a rare but severe presentation characterized by widespread microthrombosis and high mortality (7–9). Obstetric morbidity, including pregnancy loss and placental insufficiency, constitutes another major clinical domain (10).

According to the 2023 ACR/EULAR classification criteria, antiphospholipid syndrome is defined by the presence of at least one qualifying clinical manifestation—arterial or venous thrombosis, microvascular involvement, pregnancy morbidity, cardiac valve disease, or thrombocytopenia—in combination with persistent antiphospholipid antibody positivity (11). Beyond classification domains, phenotypic analyses have demonstrated that APS comprises distinct clinical patterns, including a cardiovascular subtype characterized by a predominance of arterial thrombosis, supporting the clinical relevance of evaluating arterial events as a specific manifestation rather than as part of a uniform thrombotic spectrum (12).

### 1.3 Pathophysiology of thrombosis in antiphospholipid syndrome

Antiphospholipid antibodies directed against β-glycoprotein I, prothrombin, or phospholipid–protein complexes activate endothelial cells, monocytes, and platelets, promoting a prothrombotic and proinflammatory state. Complement activation further amplifies these processes, consistent with a “two-hit” model of thrombosis (13). Persistently positive lupus anticoagulant, particularly in triple-positive profiles, remains the strongest laboratory predictor of thrombotic risk, whereas the clinical significance of novel antibodies is less clearly established (14).

At the cellular level, antibody-mediated activation induces endothelial adhesion molecule expression, increased tissue factor synthesis, and platelet activation, resulting in a sustained procoagulant milieu with inflammatory amplification (15). Despite advances in understanding these mechanisms, the relative contribution of genetic, immunologic, and environmental factors to antiphospholipid syndrome-related thrombosis remains incompletely elucidated (16, 17).

### 1.4 Role of genetics in symptomatic antiphospholipid syndrome

Genetic susceptibility has long been implicated in antiphospholipid syndrome, with the most consistent evidence involving the HLA system. Multiple HLA class II alleles have been associated with the presence of antiphospholipid antibodies in both primary antiphospholipid syndrome and antiphospholipid syndrome secondary to systemic lupus erythematosus, supporting a role for immunogenetic regulation in disease predisposition (18–21). These associations appear largely independent of specific clinical phenotypes and are relatively consistent across ethnic groups (20).

Beyond HLA-related susceptibility, classical variants such as Factor V Leiden and the G20210A prothrombin mutation, as well as polymorphisms in genes regulating fibrinolysis and homocysteine metabolism, were reported in earlier association studies, despite their subsequently questioned clinical relevance (23). Overall, antiphospholipid syndrome appears to arise from a complex interaction between immunogenetic susceptibility and additional genetic or environmental modifiers influencing clinical expression.

### 1.5 Genetic risk factors in arterial antiphospholipid syndrome

Arterial thrombosis in antiphospholipid syndrome represents a clinically distinct manifestation that has prompted the identification of genetic factors potentially modifying arterial thrombotic risk beyond the autoimmune process itself (11).

Although platelet glycoprotein polymorphisms have not shown consistent associations with arterial thrombosis, double heterozygosity for platelet glycoproteins Ia/IIa and IIb/IIIa has been linked to increased risk of arterial thrombosis and arteriosclerosis in APS secondary to SLE patients (12). In addition to polymorphisms in platelet glycoproteins, specific genes have been implicated in the development of APS and its thrombotic complications:

HLA alleles: MHC class II alleles, particularly HLA-DR and HLA-DQ, influence the presence of autoantibodies like aPL, which are central to the APS clinical phenotype (24).

Prothrombin G20210A mutation: The role of this mutation in APS-related thrombotic events remains unclear, despite its known association with venous thrombosis in other populations (23).

TNF-alpha G238A: This polymorphism has been linked to arterial thrombosis in APS patients, underscoring the importance of inflammatory pathways in APS pathogenesis (24).

β2-Glycoprotein I gene polymorphisms: Variations in the β2GPI gene, particularly G796T and G1004C, are associated with the production of aPL, contributing to the autoimmune response in APS (11).

Epigenetic influences on thrombosis in APS: Recent studies highlight the role of epigenetics in modulating thrombosis risk in APS. Antiphospholipid antibodies induce genomic and epigenetic alterations, particularly affecting monocytes, thereby promoting a prothrombotic state. Two microRNAs (miR-19b and miR-20a) have been identified as key modulators of tissue factor expression, a central initiator of coagulation and thrombosis (24). These microRNAs are released into circulation, acting as intercellular communicators, and their expression may serve as potential biomarkers for cardiovascular involvement in APS.

### 1.6 Why this review is important

Arterial antiphospholipid syndrome is associated with substantial morbidity and mortality, and its management is complicated by the need to disentangle immune-mediated mechanisms from inherited genetic susceptibility, which may represent a distinct and potentially addressable component of thrombotic risk. (13, 14). Despite extensive investigation, the contribution of inherited genetic polymorphisms to arterial thrombosis in antiphospholipid syndrome remains unclear and inconsistently reported.

This systematic review aims to critically evaluate and synthesize published evidence on genetic polymorphisms associated with arterial thrombotic manifestations in primary antiphospholipid syndrome. By assessing the consistency, quality, and limitations of existing studies, this review seeks to clarify current knowledge gaps and to generate hypotheses for future research, including improved phenotypic stratification and risk assessment in arterial antiphospholipid syndrome.

### 1.7 Objectives

#### 1.7.1 Primary objective

To systematically identify inherited genetic polymorphisms reported in association with arterial thrombosis in patients with primary antiphospholipid syndrome, with a focus on variants involved in hemostatic and immune-related pathways.

#### 1.7.2 Secondary objectives

Secondary objectives were prespecified in the registered protocol and included clinically relevant outcomes such as arterial territory, recurrence, survival, gene–environment interactions, and population or ethnic variability. However, given the limited number of eligible studies, small sample sizes, lack of replication, and substantial heterogeneity across study designs and populations, these objectives could not be addressed through systematic or quantitative analyses. They were therefore approached descriptively and narratively, as allowed by the available data.

Specifically, the secondary aims of this review were to:

- Descriptively summarize reported arterial territories involved in thrombotic events across included studies, without formal comparative analysis.
- To qualitatively and narratively synthesize reported associations between inherited genetic variants involved in hemostatic and immune-related pathways (including candidate polymorphisms such as Factor V Leiden, G20210A prothrombin mutation, and other platelet- or coagulation-related genes) and arterial thrombotic manifestations in antiphospholipid syndrome, without formal quantitative analysis.
- Summarize reported findings on population- or ethnicity-related variability where available, acknowledging the lack of structured comparative analyses and the heterogeneity of study populations.
- Report effect estimates for genetic variants when provided in individual studies, without pooling or meta-analysis.
- Narratively describe recurrence and survival outcomes when reported, recognizing that these endpoints were inconsistently assessed and do not allow systematic comparison.

## 2 Methods

This systematic review was conducted in accordance with PRISMA guidelines and followed a prespecified protocol registered in PROSPERO (CRD42024603974).

PICO format:

Population: Patients diagnosed with primary antiphospholipid syndrome with the clinical manifestation of arterial thrombosis.

Exposure: Genetic polymorphisms related to immune function, coagulation, and inflammatory responses; single nucleotide polymorphisms (SNPs).

Comparators: Healthy controls or individuals with other autoimmune diseases; antiphospholipid syndrome subgroups (e.g., obstetric, venous thrombotic antiphospholipid syndrome, antiphospholipid syndrome secondary to SLE); individuals with antiphospholipid syndrome and arterial thrombotic episodes versus those without arterial involvement.

Outcomes: Reported associations between genetic variants and arterial thrombotic manifestations.

### 2.1 Eligibility criteria – types of studies

We included observational studies (case–control and cohort designs), including genome-wide association studies (GWAS), that investigated inherited genetic polymorphisms in patients with antiphospholipid syndrome. Eligible studies assessed genetic variants in antiphospholipid syndrome patients compared with control populations and reported arterial thrombotic outcomes.

Genetic variants were not restricted to currently accepted thrombophilias but included variants historically or repeatedly investigated in antiphospholipid syndrome populations, such as Factor V Leiden, G20210A prothrombin mutation, antithrombin-related variants, MTHFR polymorphisms, and other candidate genes involved in hemostatic or immune pathways, provided arterial thrombosis outcomes were reported.

### 2.2 Eligibility criteria – exclusion criteria

Experimental studies, animal models, non-genetic investigations, narrative reviews, editorials, and conference abstracts were excluded. Studies based exclusively on transcriptomic, gene-expression, or epigenetic analyses were excluded from the genetic association synthesis, as these approaches do not assess inherited genetic susceptibility.

### 2.3 Types of participants

Studies enrolling patients with primary antiphospholipid syndrome were eligible. Studies including mixed primary and secondary antiphospholipid syndrome populations were considered only when arterial thrombotic outcomes were extractable or when primary antiphospholipid syndrome constituted the predominant subgroup. Studies lacking arterial-specific outcome data were excluded from the genetic association synthesis.

### 2.4 Types of genetic variants to be studied

Genetic variants were selected based on their evaluation in published studies reporting arterial thrombotic manifestations in antiphospholipid syndrome, rather than on their current classification as established thrombophilias. Accordingly, variants such as PAI-1 4G/5G and MTHFR polymorphisms were included to reflect the scope of the existing literature, despite their limited and controversial clinical relevance. Their inclusion does not imply pathogenic validity, and their uncertain or obsolete role is explicitly discussed.

Although HLA alleles play an important role in antiphospholipid syndrome susceptibility and immune regulation, studies assessing HLA associations predominantly focus on disease predisposition or autoantibody profiles rather than arterial thrombotic outcomes. As arterial thrombosis was a core inclusion criterion of this review, HLA association studies were not included in the genetic synthesis.

Studies based exclusively on gene-expression profiling, transcriptomic signatures, or epigenetic regulation were excluded from the genetic determinants analysis, as these approaches reflect dynamic immune activation rather than inherited genetic susceptibility.

### 2.5 Type of outcomes measured

The primary outcome was the presence of arterial thrombosis in patients with primary antiphospholipid syndrome.

Secondary outcomes prespecified in the protocol included arterial territory, recurrence, survival, population or ethnic variability, and potential gene–environment interactions. However, these outcomes were inconsistently reported across studies and were therefore addressed descriptively where available.

### 2.6 Statistical analysis

Quantitative synthesis and meta-analysis were prespecified in the study protocol, with the intention to calculate odds ratios (ORs), risk ratios (RRs), hazard ratios (HRs), genotype- or allele-based odds ratios, and attributable risk measures (AR/PAR), if sufficient and sufficiently homogeneous data were available.

However, due to the limited number of eligible studies, small sample sizes, lack of replication, inconsistent outcome definitions, and substantial heterogeneity across study designs and populations, quantitative synthesis was not feasible. Consequently, no meta-analysis was performed. Effect estimates are reported narratively as provided in individual studies, without pooling or formal comparative analysis.

### 2.7 Search methods for identification of studies

#### 2.7.1 Electronic databases

MEDLINE, Cochrane Library, ClinicalTrials.gov, and other databases for studies on genetics in antiphospholipid syndrome.

#### 2.7.2 Search strategy

Combine terms like “Antiphospholipid Syndrome,” “genetics,” “thrombophilia,” “Factor V Leiden,” “prothrombin,” “HLA alleles,” and “CD40 ligand polymorphisms.”

One reviewer (AP) independently developed the search strategy and identified all potentially eligible studies for inclusion. Rayyan^®^ software was used during the study selection process. Two review authors (SS and LG) independently screened the full texts, identified studies for inclusion, and recorded reasons for exclusion of ineligible studies. Full-text reports of all potentially relevant articles were retrieved. Any disagreements were resolved through discussion or, when necessary, by consultation with a third review author (CG). Duplicates were identified and excluded, and multiple reports of the same study were collated; individual studies, rather than individual reports, constituted the units of interest in this review. The study selection process was recorded in sufficient detail to complete a PRISMA flow diagram.

### 2.8 Data extraction

Two independent reviewers extracted data on participant characteristics, genetic variants, outcomes (e.g., incidence of thrombosis, pregnancy loss), study funding, and study design using RevMan software. Two review authors (CG and SS) independently performed data extraction, compared the extracted data, and resolved discrepancies through discussion. Unresolved disagreements were adjudicated by a third review author (AP). When necessary, the authors of the included studies were contacted to obtain additional information or clarification.

## 3 Results

### 3.1 Characteristics of eligible studies

**Figure 1:**
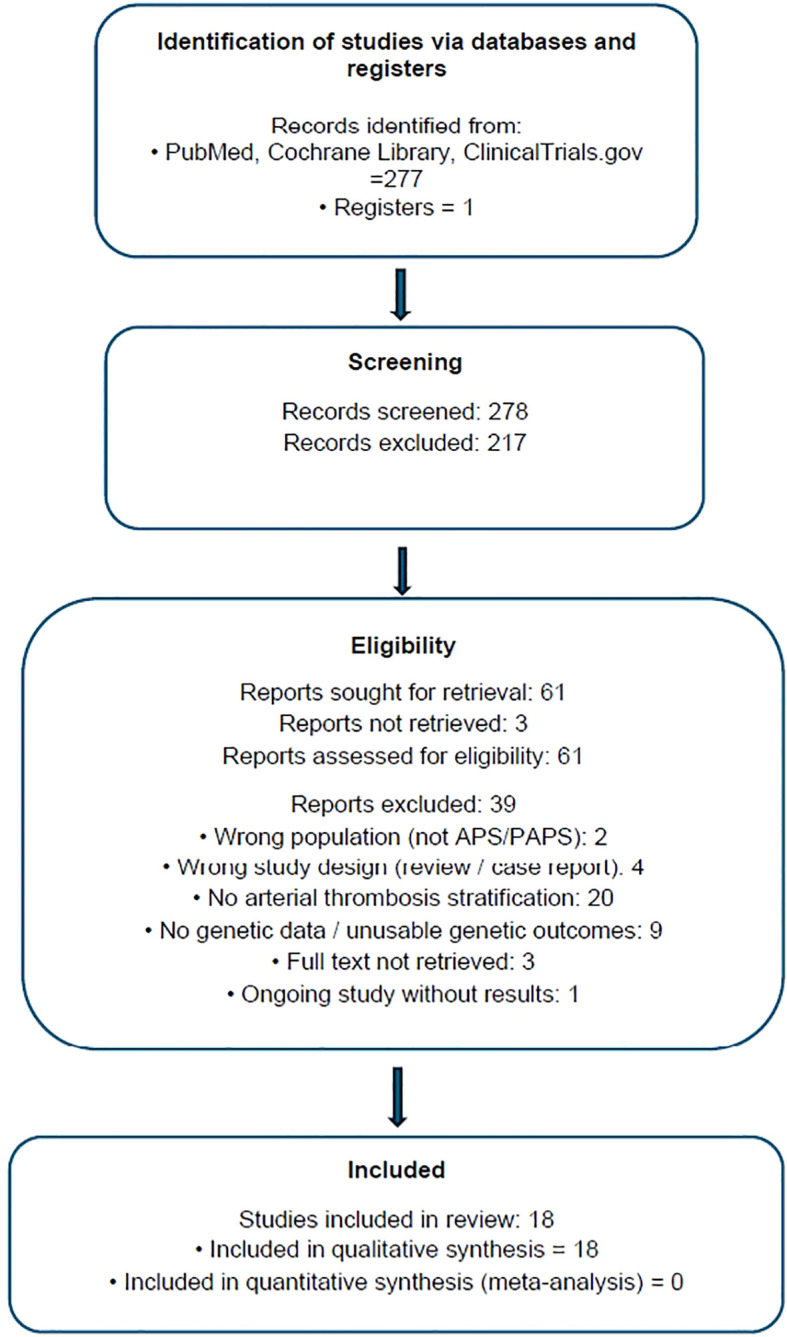
PRISMA 2020 genetic determinants of arterial thrombosis in primary antiphospholipid syndrome.

Of the 18 eligible studies initially identified, 17 investigated inherited genetic polymorphisms and were included in the genetic association synthesis. One transcriptomic study was excluded from the genetic synthesis and is discussed separately as mechanistic context (24). From 278 records screened, 61 full texts were assessed, and 18 studies met the eligibility criteria; 39 full texts were excluded for predefined reasons, including lack of arterial stratification and absence of usable genetic data. Three studies (1–3) were not included due to absence of access to the full-text article, despite institutional and interlibrary access attempts. These studies met all predefined eligibility criteria: human cohort or case–control design, assessment of genetic polymorphisms, and inclusion of antiphospholipid syndrome patients with arterial thrombotic events. Antiphospholipid syndrome was diagnosed based on the Sydney or Sapporo criteria in all included studies. Studies without stratification for arterial thrombosis or without antiphospholipid syndrome -confirmed participants were excluded or included only in qualitative synthesis if relevant data could not be extracted. (**Figure 1**. Prisma diagram).

#### 3.1.1 Hemostatic and thrombophilic gene polymorphisms

Several studies evaluated classical thrombophilic mutations:

PAI-1 4G/5G: Investigated in 4 studies (4–7). PAI-1 4G/5G showed heterogeneous findings. One Catalan antiphospholipid syndrome cohort (5) reported a strong association between the 4G allele and arterial thrombosis (OR ~6), whereas three other studies in Russian, Japanese, and Chinese Han populations did not confirm this effect. These divergent results suggest population-specific signals rather than a consistent association.

MTHFR C677T: Included in 3 studies (7–9). A strong association with arterial thrombosis was observed in one Chinese Han cohort (OR ≈ 10) (7), while another study (9) suggested a large unadjusted effect (OR ≈ 14) but at very high risk of bias; the remaining cohorts reported non-significant trends. Overall, evidence for an arterial association is inconsistent and heavily confounded.

Factor V Leiden (G1691A): Assessed in 5 studies (7, 8, 10–12), Kassis et al. evaluated APC resistance but did not report genotype-level data (9). Its association with arterial thrombosis was inconsistent; most studies showed no significant increase in arterial risk, while one reported a modest effect in combined analyses.

Prothrombin G20210A: Reported in 3 studies (7, 11, 12). Like FVL, it was associated with venous thrombosis but not consistently with arterial thrombosis in antiphospholipid syndrome (11, 12) or the mutation was absent in the Chinese Han population (7).

Factor XIII Val34Leu: Studied by Diz-Kucukkaya (13) and de la Red (14). No consistent protective or risk effect was demonstrated for arterial events, though one study reported a protective effect in high-fibrinogen subgroups.

Antithrombin and fibrinogen gene variants: Assessed by Tang (7), though results were incompletely reported.

#### 3.1.2 Platelet membrane glycoproteins

GPIa C807T and GPIIb/IIIa (PlA1/2): Evaluated in Jiménez (15) and Yonal (16) studies. GPIa 807T and double heterozygosity (807T + PlA2) were associated with increased arterial thrombosis risk (OR 3.6–4.8). The GPIbα Kozak polymorphism showed a strong association in a single small cohort (OR >10) (16), but this finding lacks external replication.

FcγRIIA (H131/R131): Sammaritano and al. reported higher thrombosis risk in H131 homozygotes (OR ~4.6; 95% CI ~1.1–19.3; p = 0.04), especially when combined with IgG2-dominant aCL subclasses (17). However, this analysis combined arterial and venous events and did not provide arterial-only estimates, limiting interpretation for arterial antiphospholipid syndrome.

#### 3.1.3 Endothelial function and immune-modulatory genes

EPCR (PROCR) haplotypes: In the study by Plasín-Rodríguez and al., neither the H1 nor the H3 haplotype showed a statistically significant association with thrombosis, and arterial events were not analyzed separately (18). Therefore, no arterial-specific inference can be drawn for this variant.

ACE I/D polymorphism: Lewis and al. found no significant association between ACE polymorphisms and arterial thrombosis in antiphospholipid syndrome (19).

CD40L, VCAM-1, ICAM-1, and others: Not consistently assessed across included studies; not eligible for synthesis based on the current dataset.

#### 3.1.4 Multi-locus and GWAS-style analyses

The multiple thrombophilia-related genes were tested in the Chinese Han cohort (7). The MTHFR T allele showed the strongest signal for arterial thrombosis (OR ~10), but data on other tested variants (e.g., PAI-1, FVL, prothrombin, fibrinogen genes) were incomplete or non-significant.

### 3.2 Risk of bias

Risk of bias across studies was variable. Most studies used validated genotyping methods and objective thrombosis diagnostics. However, the overall quality was limited by:

- Lack of adjustment for confounding variables (especially cardiovascular risk factors and treatment status).
- Incomplete stratification by antiphospholipid syndrome subtype (primary *vs*. secondary antiphospholipid syndrome).
- Selective reporting or absence of effect estimates (e.g., ORs, HRs) in several studies.
- Single-center designs with ethnically homogeneous populations, limiting generalizability.

### 3.3 Thrombosis susceptibility

The 17 included studies investigated the association between specific genetic polymorphisms and susceptibility to thrombosis in patients with antiphospholipid syndrome, with a focus on arterial thrombosis. Eighteen genes were evaluated. The results are categorized below by the type and site of thrombotic event and by associated genetic profile, emphasizing outcomes relevant for primary antiphospholipid syndrome where available. Meta-analysis was not performed for any variant due to heterogeneity and incomplete reporting.

#### 3.3.1 Genetic risk of arterial thrombosis

Several polymorphisms demonstrated a significant or suggestive association with increased arterial thrombosis risk in antiphospholipid syndrome populations:

GPIa 807C/T and GPIIb/IIIa PlA2 (15):

TT genotype of GPIa 807 and co-carriage with PlA2 increased risk of arterial thrombosis.

OR = 3.59 for 807TT alone; OR = 4.84 for combined 807T + PlA2.

MTHFR C677T (7–9):

In Chinese Han antiphospholipid syndrome patients, T-allele carriers had an increased risk of arterial thrombosis (OR = 10.27), and in an Italian cohort (8), 80% of patients with arterial thrombosis carried at least one T allele.

PAI-1 4G allele (4–7):

Although the 4G allele was strongly associated with arterial thrombosis in the Tàssies cohort (5) (OR = 5.96), this signal was absent in three other studies, including two with multivariable analyses, indicating very low and inconsistent evidence. It was associated with higher plasma PAI-1 levels, particularly in patients with antiphospholipid syndrome secondary to SLE or combined thrombosis. Overall, evidence for PAI-1 4G/5G is very low and dominated by a single outlier.

GPIbα Kozak polymorphism (16):

Strong association with arterial thrombosis in antiphospholipid syndrome patients (OR ~13). The TC genotype was overrepresented in arterial *vs*. venous cases and *vs*. healthy controls.

FcγRIIA-H131 homozygosity (17):

In the only study providing estimates, the H131 homozygous genotype was associated with increased thrombosis risk (OR ≈ 4.6), though confidence intervals were wide. IgG2 aCL predominance was also linked to a thrombotic phenotype, but on a combined dataset of arterial and venous events.

PON1 Q192R (8):

High prevalence of the R allele was observed among antiphospholipid syndrome patients with arterial thrombosis, and in the only study providing effect estimates, the RR genotype was associated with increased arterial risk (OR ≈ 7).

ACE I/D polymorphism (19):

More frequent, but not statistically significant association with arterial thrombosis: DD genotype (8/25) *vs*. non-DD (5/26), p = 0.35.

β2GPI Val247Leu (20):

More frequent arterial thrombosis in primary antiphospholipid syndrome with β2GPI Val/Leu or Leu/Leu *vs*. Val/Val: 9/24 *vs*. 5/21, but not statistically significant; OR = 1.575 (95% CI 0.416–5.960).

#### 3.3.2 Negative or null associations with arterial thrombotic risk in primary antiphospholipid syndrome

Certain polymorphisms were not associated with increased arterial thrombosis risk in antiphospholipid syndrome, or only showed association with venous thrombosis:

Factor V Leiden (7, 8, 10–12) and Prothrombin G20210A (9, 11, 12):

Weak or no association with arterial events. FVL was associated with venous thrombosis (OR ~4.0) (9), not arterial events.

FXIII Val34Leu (13, 14):

No consistent protective or risk effect for arterial thrombosis. A possible fibrinogen-dependent protective effect was reported for overall thrombosis, but no arterial-specific data were available.

tPA I/D and PAI-1 4G/5G (4):

No independent effect on arterial thrombosis in either Japanese or Caucasian antiphospholipid syndrome patients after multivariable adjustment.

PROCR H1 haplotype (18):

No arterial-specific association could be demonstrated because arterial events were not analyzed separately. No arterial-specific association was demonstrated due to lack of separate arterial subgroup analysis.

#### 3.3.3 Mixed outcomes and gene–environment interactions

Hyperhomocysteinemia (9, 12):

Significantly associated with arterial thrombosis in antiphospholipid syndrome (OR = 4.90). The effect was potentiated when combined with aPL positivity.

Site-specific thrombosis and recurrence:

Most studies reported arterial thrombosis as a composite outcome (stroke, myocardial infarction, peripheral arterial disease). Only a few (8, 15) provided granular data per arterial site. The use of composite arterial outcomes (stroke, MI, peripheral arterial disease, retinal or renal artery events) likely increased heterogeneity and may obscure site-specific genetic associations.

Only one study (8) tracked mortality and recurrence, finding that arterial thrombosis and age were independent predictors of mortality. Fibrinogen level (FNG) and DRVVT ratio were the strongest predictors of poor outcome.

No included studies systematically analyzed recurrence of arterial thrombosis by genotype beyond descriptive findings.

A concise summary of the genetic variants evaluated and their reported associations with arterial thrombosis is provided in **Table 1**, while detailed study-level results are presented in **Supplementary Table S1**.

**Table 1:** Study-level results of genetic variants evaluated in relation to arterial thrombosis in primary antiphospholipid syndrome.

Genetic pathway	Genetic variants	Key findings for arterial thrombosis	Studies reporting arterial data (first author)	Certainty of evidence
Classical thrombophilia	Factor V Leiden; Prothrombin G20210A	No consistent association with arterial thrombosis; effects mainly limited to venous events or mixed outcomes	Ames; Chopra; Danowski; Montaruli; Tang	Very low
Fibrinolysis and clot stability	PAI-1 4G/5G; FXIII Val34Leu	Conflicting and non-replicated findings; no reproducible arterial-specific signal	Aisina; Tang;Tàssies; Yasuda; de la Red; Diz-Kucukkaya.	Very low
Homocysteine metabolism	MTHFR C677T	Recurrent associations with arterial thrombosis in small cohorts; closely linked to hyperhomocysteinemia and largely unadjusted	Ames; Kassis; Tang	Very low
Platelet adhesion and activation	GPIa C807T; GPIbα Kozak variants; combined GPIa C807T + GPIIb/IIIa PlA2	Most frequently reported arterial associations; biologically plausible but derived from small, single-center studies without independent replication	Jiménez; Yonal	Low
Endothelial and anticoagulant pathways	PROCR (EPCR) H1/H3; ACE I/D; PON1 Q192R	Isolated or non-significant signals; PROCR H1 suggested protection in one study only	Plasín-Rodríguez; Lewis; Ames	Very low
Immune and autoantigen-related variants	β2GPI Val247Leu; FcγRIIA H131/R131	No statistically significant or arterial-specific association supported by consistent evidence	Pernambuco-Clímaco; Sammaritano	Very low
Transcriptomic signatures*	Interferon-regulated gene modules	Associated with thrombotic APS phenotypes (predominantly venous); reflect immune activation, not inherited genetic susceptibility	Verrou	Not applicable

*Transcriptomic and gene-expression studies are reported for mechanistic context only and were not included in the genetic association synthesis.

## 4 Discussion

### 4.1 Summary of principal findings

This systematic review identified eighteen observational studies, of which seventeen were included in the genetic association synthesis of inherited polymorphisms related to arterial thrombosis in primary antiphospholipid syndrome. Across these publications, eighteen distinct variants were assessed, representing coagulation, fibrinolysis, homocysteine metabolism, platelet adhesion and activation, endothelial pathways, and immune-inflammatory mechanisms. Most studies were small, single-center investigations involving predominantly Caucasian or East Asian populations. Study heterogeneity—particularly inconsistent reporting of arterial versus venous events and the frequent combination of primary and secondary antiphospholipid syndrome — limited comparability, and prevented quantitative synthesis.

### 4.2 Characteristics of the genetic variants studied

#### 4.2.1 Coagulation and thrombophilia-related variants

The lack of consistent arterial associations observed for classical venous thrombophilias, such as Factor V Leiden and the prothrombin G20210A mutation, supports the concept that mechanisms driving arterial thrombosis in primary antiphospholipid syndrome differ from those underlying venous thromboembolism. This distinction underscores the limited applicability of traditional thrombophilia paradigms to arterial events in antiphospholipid syndrome and suggests that arterial risk in this setting is more closely linked to immune-mediated and platelet-driven pathways than to inherited coagulation defects.

#### 4.2.2 Fibrinolysis and clot-stability pathways

Variants affecting fibrinolysis were primarily evaluated through PAI-1 4G/5G and FXIII Val34Leu.

The inconsistent findings reported for the PAI-1 4G/5G polymorphism across studies, combined with small sample sizes and lack of independent replication, argue against a robust association with arterial thrombosis in primary antiphospholipid syndrome. The historical interest in this variant likely reflects earlier hypotheses regarding fibrinolytic imbalance rather than a reproducible arterial risk factor. Overall, the available evidence does not support a clinically meaningful role for PAI-1 4G/5G in arterial thrombotic manifestations of antiphospholipid syndrome.

The FXIII Val34Leu variant, which has been hypothesized to influence fibrin cross-linking and clot stability, does not appear to exert a consistent protective or harmful effect in arterial APS. The absence of reproducible signals across studies further limits its relevance as a genetic determinant of arterial thrombosis.

#### 4.2.3 Endothelial and anticoagulant pathways

Genetic variation related to endothelial homeostasis and oxidative balance, specifically the PON1 Q192R variant, has been reported only in descriptive studies, without independent or arterial-specific effect estimates, which precludes meaningful interpretation of its role in arterial thrombosis. Beyond thrombosis-specific associations, impaired antioxidant pathways—reflected by reduced paraoxonase-1 (PON1) activity and function—have been implicated as permissive factors for antiphospholipid antibody production. Reduced PON1 activity has been reported in APS and aPL-positive patients, and common PON1 polymorphisms, including Q192R and L55M, have been described in autoimmune APS contexts, although without arterial-territory effect estimates (21, 22). Metabolic dysregulation related to transaldolase (TALDO1) deficiency has been shown to promote aPL production through secondary impairment of PON1 secretion and increased oxidative modification of phospholipid antigens (23).

The ACE I/D polymorphism, despite its known role in vascular remodeling and blood pressure regulation, did not demonstrate a significant association with arterial thrombosis in antiphospholipid syndrome. The small sample size and the fact that the observed difference was numerically higher but non-significant raise the possibility of type II error.

Neither H1 nor H3 showed a significant arterial-specific association; the protective effect of H1 applied only to overall thrombosis.

#### 4.2.4 Platelet adhesion and activation pathways

Several platelet-related genetic variants have been recurrently investigated in antiphospholipid syndrome, reflecting biologically plausible hypotheses linking platelet activation to arterial thrombosis. However, the available evidence is largely derived from small, single-center studies with heterogeneous designs and without independent replication. Consequently, these findings should be interpreted cautiously and regarded as exploratory rather than confirmatory.

#### 4.2.5 Immune and autoantigen-related variants

The β2GPI Val247Leu polymorphism was numerically more frequent in arterial cases, but the association was not statistically significant and based on small, unadjusted datasets. This variant did not reach statistical significance in arterial subsets, and current data are insufficient to support any biological or clinical effect on arterial thrombosis risk.

A transcriptomic analysis identified an interferon-regulated gene (IRG) signature associated with thrombotic primary antiphospholipid syndrome. However, differential expression was more prominent in venous than arterial thrombosis. Because these findings reflect gene expression rather than inherited polymorphisms, they are not directly comparable to SNP-based data. Transcriptomic signatures cannot be interpreted as inherited risk factors and are not comparable to SNP-based findings.

#### 4.2.6 Summary of genetic signals

Taken together, the genetic literature addressing arterial thrombosis in primary antiphospholipid syndrome is characterized by small study populations, heterogeneous designs, inconsistent outcome definitions, and a lack of independent replication. Most reported associations arise from isolated studies and should therefore be regarded as hypothesis-generating rather than confirmatory. These limitations preclude firm conclusions regarding specific genetic determinants of arterial thrombosis and highlight the need for adequately powered, multicenter studies with standardized arterial outcome assessment. See **Table 1** and **Supplementary Data**.

### 4.3 Overall completeness and applicability of evidence

The available evidence covers a narrow spectrum of antiphospholipid syndrome populations, mostly Caucasian and East Asian cohorts, with the limitation of absent explicit data in the majority of the studies (6, 9–11, 13, 15, 18, 20). There are 18 mutations, and for each mutation 1 to a maximum of 5 studies, without enough data to conduct a meta-analysis for the majority of variants. Some studies assessed multiple variants. The populations often mixed primary and secondary antiphospholipid syndrome.

The inclusion criteria were Sydney, ACR, or Sapporo. Data on recurrence, mortality, and site-specific arterial events were sparse. Few studies adjusted for conventional cardiovascular risk factors, limiting the generalizability of genetic associations. No genome-wide studies with adequate power have yet confirmed candidate polymorphisms specific to arterial antiphospholipid syndrome.

### 4.4 Quality of the evidence

The quality of evidence was low, with the predominant study design being cohort or case–control, small, single-center studies, with no randomization and limited control for confounding.

Risk of bias was high or very high in 7 of the studies (table results); 9 were rated as having moderate risk (4, 5, 7, 8, 11, 13–15, 24), one was low to moderate (12), and only one had low risk of bias (16).

Inconsistency was graded as not serious in many studies, with results internally consistent, direction of effects coherent across analyses, and biologically plausible, but with the limitation of absence of external replication with other studies. Clear discordance was observed for PAI-1 4G/5G (Catalonia positive *vs*. UK/Japan null; Russia positive) and FXIII Val34Leu (context-dependent effect at high fibrinogen only).

Indirectness was generally not serious where antiphospholipid syndrome and arterial thrombosis were explicit; it was serious when studies did not isolate arterial outcomes (e.g., FVL, β2GPI polymorphism). Imprecision was high due to low event rates with small populations and only small genotype analyses.

Publication bias was considered low, with no conflicts of interest presented.

Overall certainty of platelet receptor variants (GPIa 807T, GPIbα Kozak) across all variants was uniformly low or very low due to small sample sizes, incomplete arterial stratification, and absence of replicated findings. FXIII Val34Leu may be protective only when fibrinogen is high, with otherwise null certainty, context-dependent—graded Very low–Low. PAI-1 4G/5G, tPA I/D, ACE I/D show no consistent association with arterial thrombosis in antiphospholipid syndrome and were graded GRADE Very low–Low. FVL, Prothrombin G20210A, β2GPI Val247Leu (either venous-only signal or no arterial endpoint) were graded Very low for arterial outcomes.

### 4.5 Potential biases in the review process

Potential sources of bias include selective publication of positive genetic findings, inconsistent diagnostic criteria prior to the 2006 Sydney revision, and absence of raw genotype counts for some polymorphisms, which limited quantitative synthesis.

Three studies meeting eligibility criteria could not be included because full texts were unavailable despite institutional and interlibrary access attempts; this may introduce a limited risk of selection bias.

Some articles were accessible only via internet-access databases, local published studies, or in local languages.

Independent screening and predefined eligibility criteria limited reviewer bias.

### 4.6 Implications for practice

The evidence is observational, with low GRADE evidence quality, so general applicability is limited. Current evidence does not justify genetic testing for routine risk stratification in antiphospholipid syndrome. Variants in platelet glycoproteins or fibrinolysis pathways may help identify high-risk patients when combined with clinical risk factors, especially in recurrent or early-onset arterial thrombosis.

### 4.7 Implications for research

This systematic review shows the need for multicenter, prospective, ethnically diverse cohorts with standardized antiphospholipid syndrome classification, adjustment for conventional cardiovascular risk, and integration of high-throughput genomic and transcriptomic approaches. Longitudinal cohorts are required to determine whether genetic variants influence recurrence or mortality beyond baseline thrombosis risk.

## 5 Conclusions

In summary, the current evidence regarding inherited genetic susceptibility to arterial thrombosis in primary antiphospholipid syndrome is limited, heterogeneous, and predominantly derived from small, single-center observational studies. While some platelet-related and fibrinolytic pathway variants have been reported in association with arterial events, these findings lack replication and remain of low or very low certainty. At present, no genetic polymorphism can be considered a validated or clinically actionable determinant of arterial thrombosis risk in primary antiphospholipid syndrome. Large, multicenter, and ethnically diverse studies with standardized arterial outcome definitions are required to clarify the potential role of inherited genetic variation in arterial antiphospholipid syndrome.

## Data Availability

The original contributions presented in the study are included in the article/**Supplementary Material**. Further inquiries can be directed to the corresponding author.
