# Supplementary material for: Genetic determinants of arterial thrombosis in primary antiphospholipid syndrome: a systematic review
Source: Front Immunol. 2026 Mar 5;17:1761613. doi: 10.3389/fimmu.2026.1761613 (PMC12999903; doi:10.3389/fimmu.2026.1761613)
Supplement: Supplementary file 1 [file Table1.docx]

| **Genetic variants** | **Study** | **Population (total, comparator, PAPS, arterial thrombosis)** | **Method** | **Outcome (arterial thrombosis in APS/PAPS with mutation vs without mutation)** | **OR** | **Risk of Bias** | **GRADE** | **Comments** |
| --- | --- | --- | --- | --- | --- | --- | --- | --- |
| Hemostatic and Thrombophilic Gene Polymorphisms | | | | | | | | |
| PAI-1 (4G/4G, 4G/5G, 5G/5G) | Aisina (1) | 78 APS (43 PAPS, 35 SLE+APS); 45 genotyped; 10 controls; 22 arterial events | 78 APS (43 PAPS, 35 SLE+APS); 45 genotyped; 10 controls; 22 arterial events. | Arterial thrombosis 4G carriers vs 5G/5G: 17/34 vs 5/9 | ≈0.9–1.1 (NS). | High risk of bias (small sample, no confounder adjustment, cross-sectional) | Low ( no consistent association) | 4G allele frequent in APS; trend more evident for venous than arterial thrombosis; Statistical power limited. |
|  | Tang(2) | 67 APL+ (41 APS, 26 carriers);  105 controls (Han Chinese). | Cross-sectional; APS per Sydney; genotyping of thrombophilia variants. | No difference in PAI-1 genotype distribution between APS and controls.  (χ² = 2.49, p = 0.29). | OR not reported. | Moderate risk of bias (confounding not controlled, cross-sectional) |  | No association of PAI-1 4G/5G with arterial or venous thrombosis; findings consistent with allele frequencies in East Asian populations. |
|  | Tàssies  (3) | 247 participants: 70 PAPS, 40 secondary APS, other aPL/SLE comparators and 100 healthy controls. | Cross-sectional; APS per revised Sapporo; PAI-1 4G/5G genotyped by PCR-BsiYI digestion | Arterial thrombosis in APS with ≥1 4G allele: 33/49 (67%) vs 3/29 (10%) in 5G/5G. | OR 5.96 (1.67–21.32); 4G/4G OR 7.51; 4G/5G OR 5.25. | Moderate (observational design, no multivariable adjustment) |  | Strong association between the 4G allele and arterial thrombosis in APS; PAI-1 antigen/activity slightly higher in 4G/4G carriers but not significant. |
|  | Yasuda (4) | Japanese cohort: 77 aPL-positive (41 APS);  UK cohort: 82 aPL-positive (76 APS); both with healthy controls. | Retrospective genetic association.  PCR for tPA I/D and PAI-1 4G/5G.  APS per 1999 criteria. | No significant association with arterial thrombosis in either population; borderline unadjusted signal in Japanese cohort 21/77, disappears after adjustment | 0.51–0.82 (not significant) | Moderate (retrospective data collection, limited control for confounders, mixed APS populations) |  | PAI-1 variant not an independent predictor of arterial or venous thrombosis; consistent across ethnicities. |
| MTHFR C677T (CC/CT/TT) | Ames (5) | 118 APS patients; 31 had arterial events (20 arterial only, 11 mixed arterial and venous. | Single-centre observational cohort; APS per Sydney criteria; PCR-based genotyping; median follow-up ~10 years. | Arterial thrombosis in TT+CT vs CC: 20/25 vs 5/25. | OR ≈ 5.7 (CI not reported). | Moderate (observational, unadjusted for confounders). | Very low. | Findings suggest a strong unadjusted association between the T allele and arterial thrombosis, but residual confounding is likely. |
|  | Kassis (6) | 67 aPL-positive patients (31 PAPS); 15 with arterial thrombosis | Prospective recruitment of aPL-positive patients with retrospective confirmation of events; MTHFR genotyping and homocysteine levels measured. | TT genotype in 8/15 (53.3%) of arterial thrombosis vs 1/14 (7.1%) in comparator group. | OR: ≈14 (derived from 8/15 vs 1/14). | High risk (small subgroups, missing genotype data, no proper adjustment for confounders) |  | Arterial risk appears closely linked to hyperhomocysteinemia and MTHFR TT genotype, but high risk of bias limits interpretability. |
|  | Tang(2) | 67 aPL-positive (41 APS, 26 asymptomatic carriers); 105 healthy controls (Han Chinese). | Cross-sectional; APS per 2006 Sydney criteria; MTHFR genotyped by PCR-RFLP; logistic regression used. | In APS with arterial thrombosis (n=15): CC 1/15, CT 12/15, TT 2/15 compared with APS without arterial thrombosis. | OR 10.27 (1.17–90.17) for CT+TT vs CC | Moderate risk of bias (cross-sectional design, limited adjustment for traditional cardiovascular risk factors) |  | Strong association between the 677T allele and arterial thrombosis in APS; no association with venous thrombosis or pregnancy morbidity. |
| Factor V Leiden (G1691A) | Ames (5) | 118 APS patients; internal genotype comparators; 31 with arterial events. | Single-center cohort; APS per Sydney criteria; PCR genotyping. | Among arterial events, 2 GA carriers vs 18 GG; | Not estimable (very small nr). | Moderate (small number of carriers, no multivariable adjustment, single-center cohort) | Very low for arterial outcome (not supported) | Only two carriers identified; both had arterial events, but numbers are too small to provide a reliable estimate. |
|  | Chopra (7) | 157 APS patients; 18 PAPS (small subgroup). | Multi-center retrospective case–control; APS per consensus criteria; FVL genotyped by PCR-RFLP | FVL approximately 15% in arterial thrombosis vs 3–4% in non-thrombotic patients in the overall cohort; PAPS-specific data not provided. | Overall cohort OR 4.9 (95% CI 1.2–19.3).* | Moderate (retrospective design, incomplete arterial/PAPS stratification, limited confounder control). |  | Suggests increased thrombosis risk overall, but lack of arterial- and PAPS-specific analyses precludes clear inference for arterial APS |
|  | Danowski(8) | 122 aPL-positive patients: 17 PAPS, 59 APS with SLE, 46 SLE with aPL but no thrombosis/pregnancy loss. | Cross-sectional cohort from a tertiary lupus center; thrombophilia assessed by functional/molecular assays; logistic regression used. | Two arterial thromboses occurred in thrombophilic patients, but no subgroup data reported specifically for Factor V Leiden. | Not estimable. | Low to moderate risk of bias (cross-sectional design, relatively good characterization, but no specific arterial analysis). |  | Arterial thrombosis was associated with hypertension and elevated homocysteine, not with inherited thrombophilia in multivariable analyses. |
|  | Montaruli(9) | 60 aPL-positive individuals: 13 with arterial thrombosis, 26 with venous thrombosis, 21 without thrombosis. | Clinical cohort; aPL tested by LAC and anticardiolipin ELISA; FVL genotyped by PCR with MnlI digestion. | In PAPS with arterial thrombosis: 0/7 were carriers; FVL mutation present in 5/26 (19%) of venous thrombosis patients. | OR not calculable (no carriers in arterial thrombosis group). | High risk (mall arterial subgroup, retrospective component, no adjusted analysis). |  | Data support an association of FVL with venous but not arterial thrombosis in aPL-positive patients. |
|  | Tang(2) | 67 aPL-positive (41 APS, 26 carriers); 105 healthy controls. | Cross-sectional; APS per Sydney; genotyping for PAI-1, FVL, prothrombin, AT, fibrinogen genes. | No carriers of the G1691A mutation detected among APS patients or controls. | Not calculable. | Moderate risk of bias, mainly due to confounding. |  | Very low or absent FVL prevalence in this East Asian cohort; no assessment of effect on arterial thrombosis possible. |
| Prothrombin G20210A | Chopra (7) | 18 PAPS (subset of 157; arterial events not fully separated). | Multicenter retrospective case–control; PCR-RFLP genotyping. | No carriers with thrombosis reported; PAPS subgroup not separately analysed. | Not estimable. | Moderate risk of bias (retrospective design, rare variant, incomplete arterial/PAPS stratification). | Very low for arterial outcomes in APS. | Prothrombin G20210A was too rare to evaluate any association with arterial thrombosis in APS |
|  | Danowski (8) | 122 aPL-positive patients: 17 primary APS; 59 APS with SLE;  46 SLE with aPL but no thrombosis /pregnancy loss (control group). | Cross-sectional; thrombophilia panel including G20210A; logistic regression. | Two arterial thromboses in thrombophilic patients. No subgroup data for the prothrombin mutation. | Not estimable. | Low to moderate risk of bias (reasonable cohort, but lack of variant-specific reporting for arterial events) |  | Arterial thrombosis was associated with hypertension and elevated homocysteine, not with genetic thrombophilia. |
|  | Tang(2) | 67 aPL(+) patients (41 APS, 26 asymptomatic aPL carriers); 105 healthy controls (Han Chinese). | Cross-sectional; multi-variant genotyping panel. | No G20210A mutation detected in any APS patient or control | Not estimable. | Moderate risk of bias (cross-sectional design, limited confounder control) |  | Absence of the mutation in this population precludes any evaluation of its association with arterial or venous thrombosis |
| FXIII Val34Leu | de la Red(10) | 72 aPL-positive patients (PAPS, APS+SLE, SLE+aPL, asymptomatic aPL) and 172 healthy controls | Single-center case–control; APS per Sydney; hereditary thrombophilias excluded; FXIII Val34Leu genotyped by allele-specific PCR | Leu34 allele frequency 0.07 in patients with thrombosis vs 0.29 in those without thrombosis (p=0.04);  arterial events not separated | Not estimable. | Moderate risk of bias (modest sample size, no arterial-specific analysis, single-center). | Low | Results suggest a possible protective effect against thrombosis overall, but implications for arterial thrombosis in APS remain unclear. |
|  | Diz-Kucukkaya(11) | 60 APS patients with thrombosis (45 PAPS) and 22 aPL-positive without thrombosis; 60 matched controls. | Case–control; APS per Sapporo; FXIII Val34Leu genotyped by PCR with Hin6I; allele/genotype frequencies compared. | Data for PAPS and for arterial vs venous thrombosis were not reported separately. | Not estimable. | Moderate risk of bias (case–control design, absence of arterial and PAPS-specific analyses). |  | No clear evidence of a protective or harmful effect of FXIII Val34Leu on arterial thrombosis in PAPS. |
| Antithrombin variants | Tang(2) | 67 aPL-positive (41 APS, 26 carriers); 105 healthy controls | Cross-sectional; APS per Sydney; genotyping for antithrombin and fibrinogen polymorphisms alongside other variants. | No antithrombin gene mutations associated with thrombosis were identified in APS patients or controls. | Not estimable. | Moderate risk of bias (cross-sectional design, confounding not fully addressed, rare variants) | Low. | No evidence of a relevant antithrombin variant in this cohort; the study confirms the rarity of such mutations in East Asian populations. |
| Platelet Membrane Glycoproteins | | | | | | | | |
| Double heterozygosity (GPIa 807T + GPIIb/IIIa PlA2) | Jiménez(12) | 131 APS patients (86 PAPS, 45 APS+SLE); 102 SLE without APS; 160 healthy controls. | Case–control study; APS per Sapporo; GPIa and GPIIb/IIIa genotyped by PCR-RFLP; logistic regression for gene–gene interactions. | *GPIa 807TT genotype associated with arterial thrombosis; double heterozygosity (807T + PlA2) increased thrombosis risk overall;* | 3.59 (1.20–10.79) for 807TT and thrombosis. *Arterial-specific OR not reported* | Moderate, due to confounding (potential confounding by cardiovascular risk factors, selective reporting of arterial outcomes). | Low. | Combined platelet glycoprotein polymorphisms appear to confer higher thrombosis risk, but arterial-specific estimates in APS are not reported. |
| Glycoprotein Ibα and Ia/IIa polymorphisms | Yonal (13) | 60 APS (all PAPS): 30 with thrombosis (10 arterial only, 11 venous only, 9 mixed), 30 without thrombosis; 63 matched controls. | Case–control genetic association; GPIa C807T genotyped by real-time PCR; GPIbα Kozak and VNTR polymorphisms by PCR + restriction digestion; clinical events imaging-confirmed. | GPIa C807TT more frequent in APS with thrombosis; GPIbα Kozak TC strongly associated with arterial thrombosis; VNTR D allele less frequent in APS, suggesting protection | TT vs CC/CT: 16.18 (0.87–301.84); GPIbα Kozak TC vs TT: 10.67 (1.39–82.07). | Low to moderate (case–control design, small sample but good phenotypic characterization and exclusion of major confounders) | Moderate. | Data support a strong association of platelet glycoprotein polymorphisms, especially GPIbα Kozak TC, with arterial thrombosis in PAPS. |
| FcγRIIA (H131/R131) | Sammaritano (14) | 30 PAPS; 30 SLE+APS; 103 healthy controls | Cross-sectional observational; APS based on clinical criteria; aCL IgG subclasses measured by ELISA; FcγRIIA genotyped by allele-specific PCR | Thrombosis (arterial ± venous) more frequent in IgG2-positive vs IgG2-negative patients; no separate OR for isolated arterial events | OR: ≈4.6 (for thrombosis in IgG2-positive vs IgG2-negative No separate OR for arterial events in PAPS | Serious risk of bias(small sample, confounding by indication, selective endpoint reporting, arterial events not isolated) | Low. | Suggests that IgG2 aCL subclass is associated with thrombotic phenotype; genetic and arterial-specific effects remain unclear. |
| Endothelial Function and Immune-Modulatory Genes | | | | | | | | |
| PROCR (EPCR) H1 (7014G/C) and H3 (1651C/G) haplotypes | Plasín-Rodríguez(15) | 83 APS (68 PAPS, 15 APS+SLE); 63 healthy control | Cross-sectional clinical study; APS per Miyakis 2006; PROCR haplotypes genotyped by qRT-PCR with melting-curve analysis. | No 2×2 data for arterial thrombosis in PAPS by haplotype; overall thrombosis not significantly associated with H1 or H3. | Not estimable for PAPS  OR ≈ 1.56 for VV+VL vs LL (non-significant in APS cohort). | Low to moderate — cross-sectional design, relatively small sample, insufficient reporting for arterial PAPS outcome. | Low. | No evidence that PROCR haplotypes modify arterial thrombosis risk in APS; V allele mainly associated with thrombocytopenia. |
| β2GPI Val247Leu (G2GP1 Val247Leu) | Pernambuco-Clímaco(16) | 45 primary APS (PAPS); no external control group. | Cross-sectional study; APS per Sapporo; β2GPI Val247Leu genotyped by PCR-RFLP; thrombosis type from clinical records. | Arterial thrombosis in PAPS carriers (Val/Leu or Leu/Leu) vs Val/Val: 9/24 vs 5/21. | OR 1.575 (95% CI 0.416–5.960), not significant. | High risk of bias (small sample size, no adjustment for confounding, retrospective outcome collection). | Low | Slightly higher frequency of arterial events in Leu carriers, but underpowered and not statistically significant. |
| ACE I/D | Lewis (17) | 51 PAPS; 42 secondary APS (SLE+APS); 117 healthy controls. | Cross-sectional; APS per Sydney criteria; ACE I/D genotyped by PCR; traditional risk factors extracted from records. | In PAPS, arterial thrombosis in DD genotype vs non-DD: 8/25 vs 5/26, p = 0.35 (NS). | OR 1.98 (95% CI 0.55–7.16). | Moderate to serious risk of bias(cross-sectional design, small PAPS subgroup, incomplete multivariable adjustment in subgroup analyses) | Low. | DD genotype showed a nonsignificant trend toward more arterial events; sample size and residual confounding limit interpretation. |
| PON1 Q192R | Ames (5) | 118 APS patients; internal genotype comparators; 31 with arterial events. | Single-center observational cohort; APS per Sydney; PON1 Q192R genotyped by PCR. | Arterial thrombosis in RR vs QR+QQ: 5/6 vs 20/29. | OR ≈ 7.1 (CI not reported). | Moderate risk of bias (observational design, very small number of RR carriers, lack of multivariable adjustment). | Low. | RR genotype was overrepresented among arterial cases, but few carriers and absence of adjustments reduce certainty. |
| Multi-locus and GWAS-Style Analyses | | | | | | | | |
| Fibrinogen variants | Tang(2) | 67 aPL(+) patients (41 APS, 26 asymptomatic aPL carriers); 105 healthy controls (Han Chinese). | Cross-sectional; genotyping of fibrinogen α, β, and γ chain polymorphisms by allele-specific PCR, alongside other thrombophilia-related variants. | No fibrinogen gene polymorphisms detected in any of the patients or controls. | Not estimable. | Moderate risk of bias(cross-sectional design, confounding not fully adjusted, absence of variants limits inference) | Low. | Confirms near-absence of these fibrinogen variants in East Asian populations; no evidence for a role in arterial thrombosis in APS. |
| Transcriptomic and Expression-Based Studies | | | | | | | | |
| Transcriptomic interferon-regulated signature* | Verrou (18) | 62 PAPS (33 with thrombosis, 29 without); 14 healthy controls. | Cross-sectional transcriptomic study; whole-blood RNAseq; differential expression and WGCNA; APS per 2006 Sydney criteria. | IRG module upregulated in thrombotic vs non-thrombotic PAPS; arterial vs venous thrombosis not analyzed separately. | Not estimable (no genotypic comparison). | Moderate risk of bias (cross-sectional design, small sample, no external validation cohort, arterial events not isolated) | Low. | Supports an interferon-driven inflammatory signature in thrombotic PAPS but does not define a specific genetic polymorphism risk for arterial events. |

*Transcriptomic and gene-expression studies are reported for mechanistic context only and were not included in the genetic association synthesis.

**Suplimentary Tabel S1 Detailed study-level results of genetic variants evaluated in relation to arterial thrombosis in primary antiphospholipid syndrome**

Bibliography

1. Aĭsina RB, Mukhametova LI, Ostriakova EV, Seredavkina NV, Patrushev LI, Patrusheva NL, et al. [Polymorphism of the plasminogen activator inhibitor type 1 gene, plasminogen level and thrombosis in patients with antiphospholipid syndrome]. Biomed Khim. 2014;60(1):72–93.

2. Tang Z, Shi H, Liu H, Cheng X, Su Y, Ye J, et al. Methylenetetrahydrofolate Reductase 677T Allele Is a Risk Factor for Arterial Thrombosis in Chinese Han Patients with Antiphospholipid Syndrome. Biomedicines. 2022 Dec 26;11(1):55.

3. Tàssies D, Espinosa G, Muñoz-Rodríguez FJ, Freire C, Cervera R, Monteagudo J, et al. The 4G/5G polymorphism of the type 1 plasminogen activator inhibitor gene and thrombosis in patients with antiphospholipid syndrome. Arthritis Rheum. 2000 Oct;43(10):2349–58.

4. Yasuda S, Tsutsumi A, Atsumi T, Bertolaccini ML, Ichikawa K, Khamashta MA, et al. Gene polymorphisms of tissue plasminogen activator and plasminogen activator inhibitor-1 in patients with antiphospholipid antibodies. J Rheumatol. 2002 June;29(6):1192–7.

5. Ames PRJ, Merashli M, Chis Ster I, D’Andrea G, Iannaccone L, Marottoli V, et al. Survival in primary antiphospholipid syndrome. A single-centre cohort study. Thromb Haemost. 2016 June 2;115(6):1200–8.

6. Kassis J, Neville C, Rauch J, Busque L, Chang ER, Joseph L, et al. Antiphospholipid antibodies and thrombosis: association with acquired activated protein C resistance in venous thrombosis and with hyperhomocysteinemia in arterial thrombosis. Thromb Haemost. 2004 Dec;92(6):1312–9.

7. Chopra N, Koren S, Greer WL, Fortin PR, Rauch J, Fortin I, et al. Factor V Leiden, prothrombin gene mutation, and thrombosis risk in patients with antiphospholipid antibodies. J Rheumatol. 2002 Aug;29(8):1683–8.

8. Danowski A, de Azevedo MNL, de Souza Papi JA, Petri M. Determinants of risk for venous and arterial thrombosis in primary antiphospholipid syndrome and in antiphospholipid syndrome with systemic lupus erythematosus. J Rheumatol. 2009 June;36(6):1195–9.

9. Montaruli B, Borchiellini A, Tamponi G, Giorda L, Bessone P, van Mourik JA, et al. Factor V Arg506-->Gln mutation in patients with antiphospholipid antibodies. Lupus. 1996 Aug;5(4):303–6.

10. de la Red G, Tàssies D, Espinosa G, Monteagudo J, Bové A, Plaza J, et al. Factor XIII-A subunit Val34Leu polymorphism is associated with the risk of thrombosis in patients with antiphospholipid antibodies and high fibrinogen levels. Thromb Haemost. 2009 Feb;101(2):312–6.

11. Diz-Kucukkaya R, Hancer VS, Inanc M, Nalcaci M, Pekcelen Y. Factor XIII Val34Leu polymorphism does not contribute to the prevention of thrombotic complications in patients with antiphospholipid syndrome. Lupus. 2004;13(1):32–5.

12. Jiménez S, Tàssies D, Espinosa G, García-Criado A, Plaza J, Monteagudo J, et al. Double heterozygosity polymorphisms for platelet glycoproteins Ia/IIa and IIb/IIIa increases arterial thrombosis and arteriosclerosis in patients with the antiphospholipid syndrome or with systemic lupus erythematosus. Ann Rheum Dis. 2008 June;67(6):835–40.

13. Yonal I, Hindilerden F, Hancer VS, Artim-Esen B, Daglar A, Akadam B, et al. The impact of platelet membrane glycoprotein Ib alpha and Ia/IIa polymorphisms on the risk of thrombosis in the antiphospholipid syndrome. Thromb Res. 2012 Apr;129(4):486–91.

14. Sammaritano LR, Ng S, Sobel R, Lo SK, Simantov R, Furie R, et al. Anticardiolipin IgG subclasses: association of IgG2 with arterial and/or venous thrombosis. Arthritis Rheum. 1997 Nov;40(11):1998–2006.

15. Plasín-Rodríguez MA, Rodríguez-Pintó I, Patricio P, Monteagudo J, Cervera R, Reverter JC, et al. The H1 haplotype of the endothelial protein C receptor protects against arterial thrombosis in patients with antiphospholipid syndrome. Thromb Res. 2018 Sept;169:128–34.

16. Pernambuco‐Climaco JM, Brochado MJF, Freitas MVC, Roselino AMF, Paulo Louzada‐Junior. Val/Leu^247^ Polymorphism of β2‐glycoprotein I in Brazilian Patients with Antiphospholipid Syndrome—A Genetic Risk Factor? Annals of the New York Academy of Sciences [Internet]. 2009 Sept [cited 2025 Nov 28];1173(1):509–14. Available from: https://nyaspubs.onlinelibrary.wiley.com/doi/10.1111/j.1749-6632.2009.04655.x

17. Lewis NM, Katsumata K, Atsumi T, Sanchez ML, Romero FI, Bertolaccini ML, et al. An evaluation of an angiotensin-converting enzyme gene polymorphism and the risk of arterial thrombosis in patients with the antiphospholipid syndrome. Arthritis Rheum. 2000 July;43(7):1655–6.

18. Verrou KM, Sfikakis PP, Tektonidou MG. Whole blood transcriptome identifies interferon-regulated genes as key drivers in thrombotic primary antiphospholipid syndrome. J Autoimmun. 2023 Jan;134:102978.
